# Rare partial octosomy and hexasomy of 15q11-q13 associated with intellectual impairment and development delay: report of two cases and review of literature

**DOI:** 10.1186/s13039-018-0365-5

**Published:** 2018-02-05

**Authors:** Haiyu Li, Juan Du, Wen Li, Dehua Cheng, Wenbin He, Duo Yi, Bo Xiong, Shimin Yuan, Chaofeng Tu, Lanlan Meng, Aixiang Luo, Ge Lin, Guangxiu Lu, Yue-Qiu Tan

**Affiliations:** 1 Institute of Reproduction and Stem Cell Engineering, Central South University, Changsha, Hunan 410078 People’s Republic of China; 2 Reproductive and Genetic Hospital of CITIC-Xiangya, Changsha, Hunan 410078 People’s Republic of China

**Keywords:** Isodicentric 15, Partial octosomy of 15q, Mental retardation, Developmental delay, Hyperpigmentation, *P* gene

## Abstract

**Background:**

Small supernumerary marker chromosomes (sSMCs) are common structurally abnormal chromosomes that occur in 0.288% of cases of mental retardation. Isodicentric 15 (idic(15)) is common in sSMCs and usually leads to a rare chromosome disorder with distinctive clinical phenotypes, including early central hypotonia, developmental delay, epilepsy, and autistic behavior. It was previously shown that the partial tetrasomy 15q and partial hexasomy 15q syndromes are usually caused by one and two extra idic(15), respectively. Karyotypes containing a mosaic partial octosomy 15q resulting from three extra idic(15) have rarely been reported.

**Case presentation:**

Two patients with profound intellectual impairment, development delay and hyperpigmentation were recruited for this study. The phenotype was relatively more severe in patient 1 than in patient 2. Conventional cytogenetic analysis of peripheral blood obtained from patients 1 and 2 revealed rare mosaic karyotypes containing sSMCs, i.e., mos 49,XX,+mar × 3[83]/48,XX,+mar × 2[7]/46,XX[10] and mos 48,XX,+mar × 2[72]/47,XX,+mar[28], respectively. The results of analyses of copy number variation (CNV) and fluorescence in situ hybridization (FISH) analyses, showed that the sSMCs were found to be idic(15) involving the Prader-Willi/Angelman Syndrome Critical Region (PWACR) genes and the *P* gene, with duplication sizes of 6.3 Mb and 9.7 Mb, respectively. DNA fingerprinting analysis of patient 1 showed a maternal origin for the idic(15). Both patients had mosaic idic(15) karyotypes: patient 1 had cells with a 15q partial octosomy (83%), and patient 2 had cells with a 15q partial hexasomy (72%).

**Conclusions:**

We detected two rare mosaic idic(15) karyotypes that were associated with congenital abnormalities, including a rare mosaic octosomy of 15q11-q13. Our cases further validate the notion that the phenotypic severity is correlated with the level of mosaicism and the dosage effect of related genes in the proximal 15q.

## Background

Small supernumerary marker chromosomes (sSMCs) can be defined as small structurally abnormal chromosomes that occur in addition to the normal 46 chromosomes [1]. Usually, an sSMC is smaller than chromosome 20 and cannot be unambiguously identified or characterized using only conventional chromosome banding techniques [2]. These chromosomes occur in 0.04% of newborns and 0.288% of cases with mental retardation [3]. Individuals who carry sSMCs present with a broad spectrum of clinical characteristics, and ranging from a normal presentation to severe birth defects [4]. The effects of sSMCs on a patient’s features are associated with their size, the presence of euchromatic material and the level of mosaicism [5].

The sSMCs that originate from chromosome 15, i.e. sSMC(15), are common and can usually be identified using molecular cytogenetic techniques. Chromosome 15 contains many low copy repeats which is prone to unequal crossover, and can form inverted duplication 15 (inv dup(15)) or special sSMC(15) named isodicentric 15 (idic(15)) which includes a chromosome fragment that is duplicated from end-to-end as a mirror image. The phenotypes of patients with idic(15) appear to be highly dependent on the breakpoint. For example, almost no clinical signs were detectable in a case with idic(15)(pter→q12), whereas clinical signs were presented in a patient with the karyotype 47,+idic(15)(pter→q13) [6]. Some researchers have proposed that an idic(15) without the PWS/AS critical region (PWACR) is clinically neutral, while an idic(15) containing the PWACR might result in severe clinical phenotypes, such as intellectual disability, development delay, autism, seizures, and behavioral problems [7–9]. Furthermore, paternally inherited idic(15) might be associated with a normal phenotype, whereas maternally inherited idic(15) is likely to result in development impairments [10].

The number of idic(15) reported across different cases has varied. Partial tetrasomy 15q, which is caused by one idic(15), is relatively common (approximately 80% of cases) [7, 8, 11–19], and partial hexasomy 15q, which is caused by one or two idic(15) or tricentric der(15), has also been occasionally described [9, 20–23]. However, individuals who carry more than two idic(15) have rarely been reported. In this study, we described two children with congenital abnormalities who carried rare types of idic(15) in mosaic forms that were identified by molecular cytogenetic techniques, including single-nucleotide polymorphism (SNP) arrays and fluorescence in situ hybridization (FISH). To improve the value of genetic counseling in affected cases, we explored the relationship between the phenotypes and karyotypes of these patients.

## Case presentation

Patient 1 was a 3-year-old Chinese girl who was born after 38 weeks of uneventful gestation to non-consanguineous healthy parents. She was the first pregnancy and first child of the parents. Her birth weight, length and head circumference were normal, but she exhibited obvious birth defects. She had a cleft palate and extensive skin hyperpigmentation at birth. Her Apgar scores were 7 and 5 at one and five minutes after birth, respectively.

**Fig. 1:**
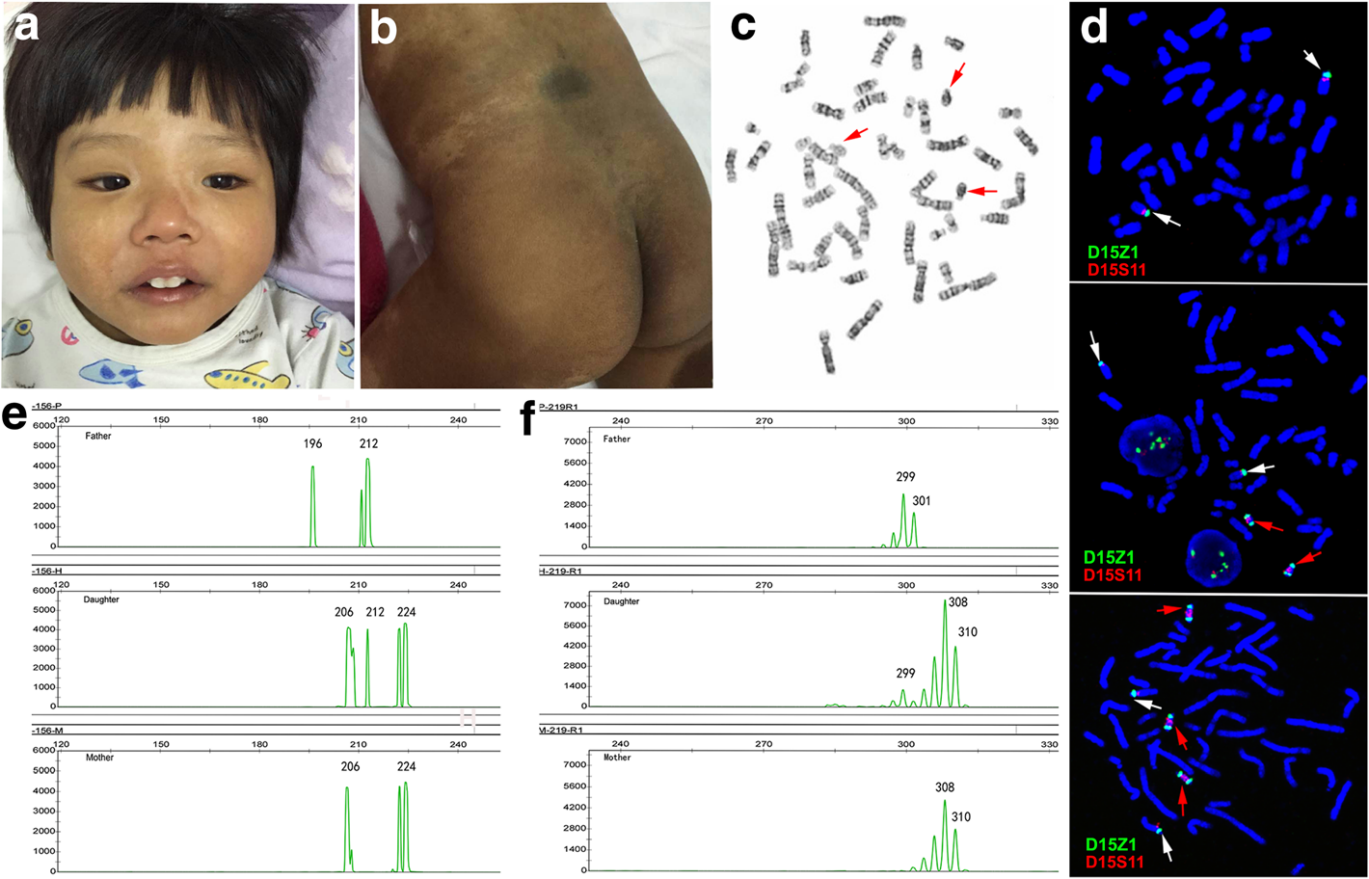
The appearance, karyotyping, FISH and DNA fingerprinting results for patient 1. **a** Frontal view of patient 1 displays some facial dysmorphism, including ocular hypertelorism, nystagmus and uneven pigmentation. **b** Deeper pigmentation on the buttocks and back. **c** Partial karyotype by G-banding showing three extra idic(15) regions (red arrow) **d** FISH analysis using D15Z1 (green arrow) and D15S11 (red arrow) showing that each idic(15) has two green hybridization signals and two red hybridization signals. A normal chromosome 15 has one green and one red hybridization signal. **e** and **f** Electropherograms of the markers D15S156 (**e**) and D15S219 (**f**) in the father, children and mother (top to bottom). The children have three different alleles of D15S156 and D15S219. **e** The informative marker of D15S156 shows two different maternal alleles (206 and 224) and one paternal allele (212), indicating that the duplicated region of patient 1 was maternal. **f** The informative marker of D15S219 shows two different maternal alleles (308 and 310) and one paternal allele (299), again illustrating that the duplicated region was maternal

Her growth was nearly normal, but she was slow to reach her developmental motor milestones. Her height, weight, and head circumference were 55.6 cm (25^th^percentile), 3900 g (< 3^rd^ percentile) and 34.2 cm (< 3^rd^ percentile), respectively, at 2 months. She began to stably raise her head at 20 months. Although she is currently 3 years old, she cannot sit, crawl, or walk independently. With the help of her parents, she can stand for a short time but tires easily. Her daily needs are completely taken care of by her family. She was severely delayed in intelligence with a development quotient (DQ) score of 20 at 20 months. She has exhibited extensive skin hyperpigmentation over her whole body, particularly in the trunk, limbs and perineum, since birth. A darker color is distributed diffusely on her back and buttocks and linearly on her arms. The light coloration on her face, neck, hands, a part of the abdominal skin and the left leg are the only areas of normal coloration on her whole body. The percentage of her body covered with hyperpigmentation is estimated to exceed 85% (Fig. 1). Other tests, such as brain magnetic resonance imaging (MRI), hearing tests, and biochemical tests, were normal.

**Fig. 2:**
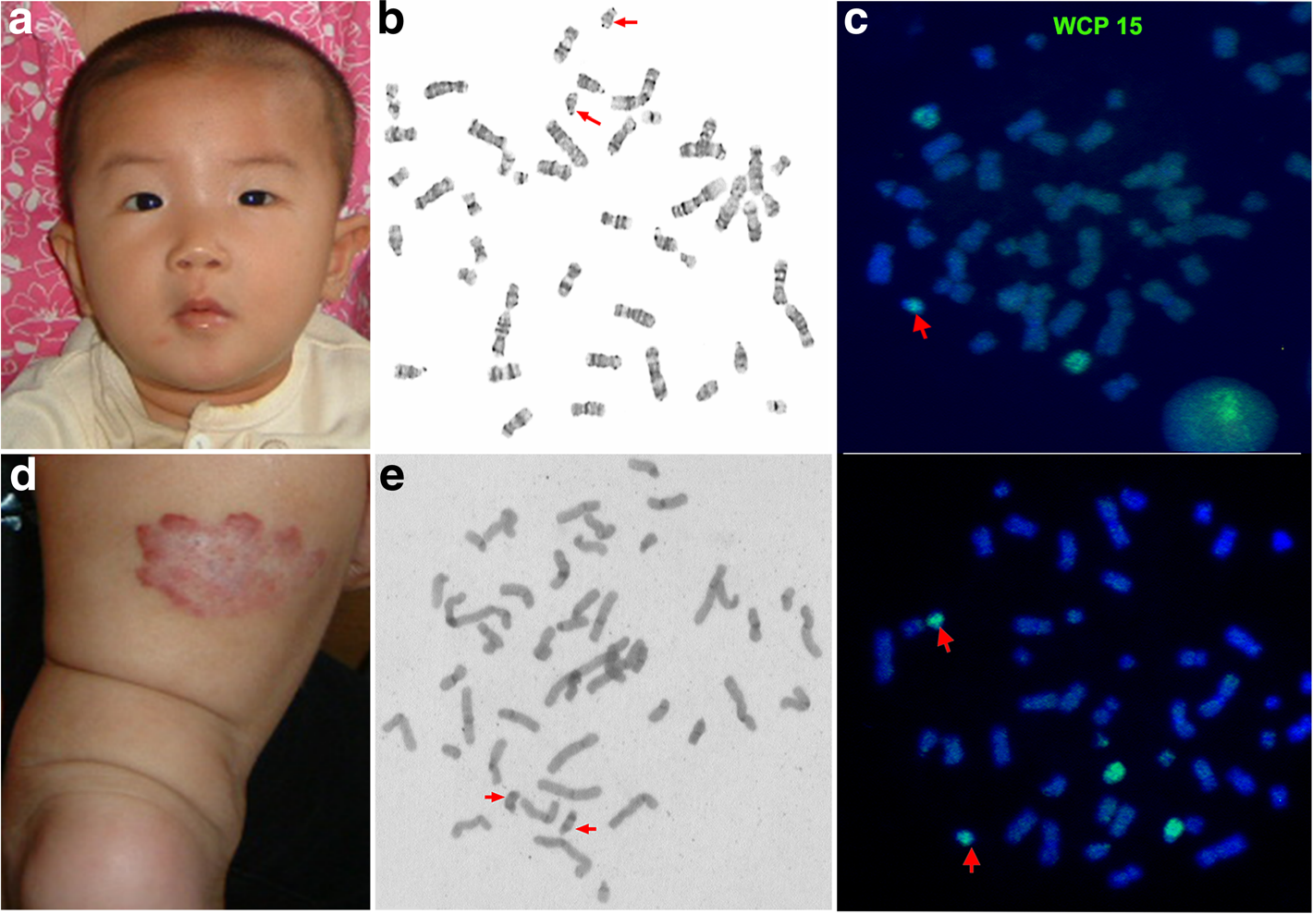
The appearance, karyotyping and FISH results for patient 2. **a** The proband has mild facial dysmorphism with low-set ears, a depressed nasal bridge and ocular hypertelorism. **b** Partial karyotype by G-banding showing two extra idic(15) regions (red arrow) **c** FISH analysis of patient 2 using whole-chromosome painting (WCP) probes showing one and two idic(15) regions **d** The patient 2 had a hemangioma (2 cm × 4 cm) on her left leg. **e** Partial karyotype by C-banding showing that each SMC(15) had two centromeres, indicating that the SMC(15) was idic(15)

Patient 2 was an eight-month-old girl. She was born after 39 weeks of uneventful gestation. She was the second pregnancy and second child of the parents, and all other members of her family were healthy. Her birth weight was 4450 g (> 97^th^percentile). Even though she is currently eight months old, she cannot turn over, sit or crawl and can only raise her head unsteadily. She was seriously delayed for 5 months in motor and intelligence with a DQ score of 29.7. The clinical phenotype of patient 2 was similar to that of patient 1, but patient 2 only had slight hyperpigmentation and no cleft palate (Fig. 2). Her brain computed tomography (CT) scan and electroencephalography (EEG) were abnormal. Her skin hyperpigmentation was also found on her neck and limbs; notably, a hemangioma (2 cm × 4 cm) on her left leg broadened the phenotypic spectrum of the 15q duplication (Fig. 2).

### Cytogenetic and molecular cytogenetic analyses

Chromosome analysis was performed by G-banded and/not C-banded metaphases in cultured peripheral blood lymphocytes. FISH analysis was performed on metaphase chromosomes in peripheral blood lymphocytes and oral mucosal cells with D15Z1 (15p11, green signal)/D15S11 (15q11.2, red signal) probes, which covered the critical region of the PWS/AS syndrome (PWACR) in the patient 1, and whole-chromosome painting (WCP) probes were used in patient 2 according to the manufacturer’s instruction. All probes were purchased from Abbott-Vysis (Downers Grove, IL, USA). Single hybridization patterns were analyzed using VideoTesT-FISH 2.1 software (Version number 5.0.74.4803, VideoTesT, Ltd., Russia). SNP microarrays were used to analyze genomic DNA that were extracted from the peripheral blood of patients using QIAamp® DNA Midi Kit (QIAGEN, Hilden, Germany). DNA concentration was measured using NanoDrop spectrophotometer (ND-1000, Thermo Fisher Scientific, USA) and DNA quality was determined by agarose gel electrophoresis (1%). For each sample, 2.0 μg of genomic DNA was used for testing which was performed according to the protocol of CytoScan750K reagent kit (Affymetrix, USA). Image scanning and data analyses were performed on Chromosome Analysis Suite (CHAS, Affymetrix, USA). The base pair positions of detected genomic imbalances were designated according to the February 2009 (GRCh37/hg19) Assembly in the UCSC Human Genome browser (http://genome.ucsc.edu).

### DNA fingerprinting analysis

Genomic DNA was extracted using the methods described above. Markers were chosen from previous studies based on the breakpoint in patient 1 [24, 25]. These markers included D15S219, D15S156, D15S1048, D15S1019, D15S1043, D15S165, and D15S184 (Table 1).

**Table 1:** DNA fingerprinting results for 7 STR loci in patient 1

	Size of the fragment		
Locus	Father	Patient 1	Mother
D15S219	(299, 301)	(299, 308/310)	(308, 310)
D15S156	(196, 212)	(212, 206/224)	(206, 224)
D15S1048	(197, 216)	(197, 220)	(220, 220)
D15S1019	(198, 201)	(199, 201)	(199, 201)
D15S1043	(87, 95)	(81, 87)	(81, 87)
D15S165	(165, 178)	(165, 178)	(165, 178)
D15S184	(223, 278)	(223, 278)	(223, 278)

## Results

**Fig. 3:**
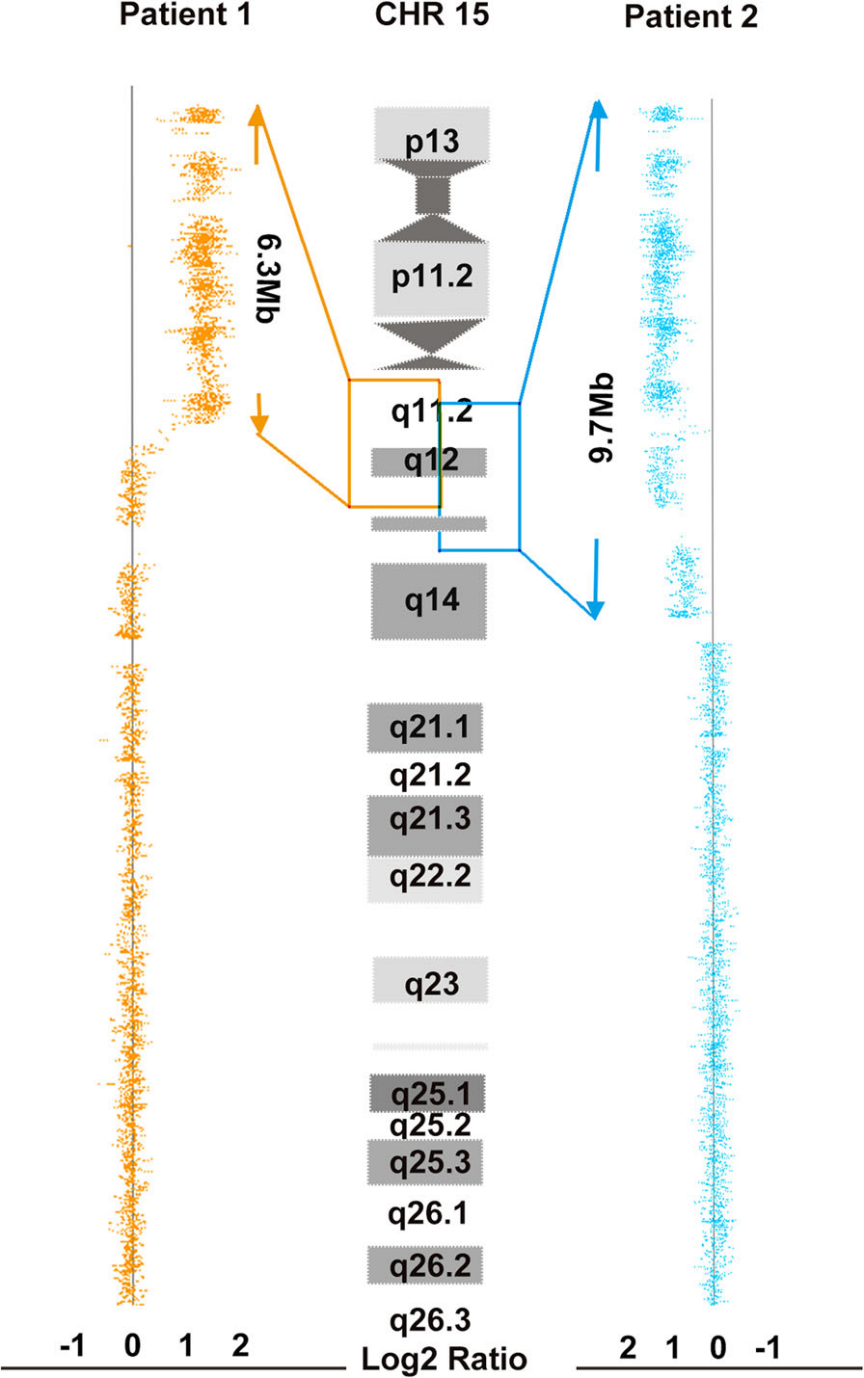
SNP array analysis of both patients. A gain in copy number in the 15q11q13 region of patient 1 (left) and patient 2 (right). The orange box shows the location of the copy number gain on chromosome 15 in patient 1, and the blue box shows the location of the copy number gain on chromosome 15 in patient 2. The copy number was beyond the maximum limit of the software (4×). The values on the X-axis represent the log2 ratios for the patients

Conventional G-banding showed that the karyotypes of the patients were mos 49,XX,+mar × 3[83]/48,XX,+mar × 2[7]/46,XX[10] and mos 48,XX,+mar × 2[72]/47,XX,+mar[28] (Figs. 1 and 2), respectively. Chromosome microarray analysis performed using Cytoscan 750 k SNP array revealed an abnormal female array profile with a copy number gain in the regions 15q11.2–13.1(22,770,421–29,073,540, 6.3 Mb) and 15q11.2–13.3 (22,770,421–32,444,043, 9.7 Mb) (Fig. 3). The results of FISH analyses confirmed that the sSMC was derived from chromosome 15 and was an idic(15) chromosome because each sSMC had two hybridization signals (Fig. 1). Therefore, patient 1 had a mosaic partial octosomy karyotype, 49,XX,+idic(15) × 3(pter→q13::q13 → pter)[83]/48,XX,+idic(15) × 2(pter→q13::q13 → pter)[7]/46,XX[10].ish idic(15)(q13.1)(D15Z1++,D15S11++).arr 15q11.2q13.1(22,770,421–29,073,540) × 4 dn (reaching the maximum of the software design), while patient 2 had a mosaic partial hexasomy karyotype, 48,XX,+idic(15) × 2(pter→q13::q13 → pter)[72]/47,XX,+idic(15)(pter→q13::q13 → pter)[28].arr 15q11.2q13.3 (22,770,421–32,444,043] × 4 dn (reaching the maximum of the software design). Similar FISH results were obtained using oral mucosal cells from patient 1, in whom the ratio of octosomy 15q cells was 85% (180/210). In addition, DNA fingerprinting analysis of patient 1 showed that the child had three different alleles at D15S156 and D15S219, respectively (Fig. 1). These informative markers revealed that one allele was of paternal origin and the remaining two distinct alleles were of maternal origin. These results suggested that in patient 1, biparental inheritance of two normal chromosome 15 s and maternal inheritance of the idic(15) had occurred, which eliminated the possibility of uniparental disomy (UPD).

## Discussion

In this study, we report two patients with serious intellectual impairment and development delay who carried high ratios of mosaic sSMCs. The sSMCs were confirmed by karyotyping, FISH and SNP arrays to be idic(15) and derived from 15pter-15q13.

The chromosome region 15q11-q13 is a potential hot spot for chromosomal duplication and deletion due to numerous interspersed and tandem segmental duplications in this region [26, 27]. Small duplications without the PWACR are usually believed to be clinically neutral [8, 28], but larger genomic rearrangements that cover the PWACR can result in three distinct neurodevelopmental disorders, namely, Prader-Willi syndrome (PWS), Angelman syndrome (AS), and 15q duplication syndrome (Dup15q syndrome) [29]. The two patients presented in this study had severe phenotypes, including cognitive disabilities, psychomotor delays, and speech and behavioral problems. They also had segment gains of 6.3 Mb and 9.7 Mb in 15q, respectively, which is consistent with previously reported cases of Dup15q syndrome [29].

Dup15q syndrome could be caused by interstitial duplication or idic(15), and patients with an interstitial duplication exhibited a milder phenotype than those with idic(15) [19]. An interstitial duplication usually comprises one extra copy of 15q11-q13, which results in trisomy of 15q11-q13. And an idic(15) typically adds two extra copies of 15q11-q13, leading to tetrasomy of 15q11-q13. Nevertheless, a single idic(15) that included four extra copies of 15q11-q13 resulting in hexasomy of 15q, has also been reported [22]. To date, different copy number variations (CNVs) of the 15q11-q13 fragment have been observed to range from 3 to 6 copies, resulting in triplication [15, 30], tetrasomy [11, 12, 16–18], pentasomy [14] and hexasomy [9, 21–23]. Tetrasomy 15q, containing one extra idic(15), is relatively common.

Battaglia reviewed the phenotypes of patients with 15q duplication and showed that tetrasomy of 15q causes a more serious phenotype than 15q triplication [31]. Therefore, more repeats are likely to result in more serious phenotypes. In our study, the symptoms of patient 1, who carried mosaic partial octosomy and hexasomy of 15q, were more severe than those observed in patient 2, who carried a mosaic partial hexasomy and tetrasomy of 15q. Compared with patient 2, patient 1 had a lower DQ and more delayed developmental milestone events. For example, patient 1 could not hold her head up at 8 months, while patient 2 could do so. The data in our study further support the above mentioned hypothesis regarding the relationship between the 15q copy number and the phenotypic severity.

In the present study, the karyotype of patient 1 was mos 49,XX,+idic(15) × 3(pter→q13::q13 → pter)[83]/48,XX,+idic(15) × 2(pter→q13::q13 → pter)[7]/46,XX[10]. To the best of our knowledge, only two similar cases have previously been reported [32, 33]. Cockwell et al. described a patient with dynamic mosaicism of inv. dup(15) [32]. Chromosome and FISH analyses showed that 35 of 50 GTG-banded metaphases had morphologically distinct sSMCs dervied from chromosome 15. Furthermore, 3 of 35 cells had 3 sSMCs, including inv. dup(15), a ring chromosome and a minute chromosome. The other case was a girl with mental retardation, who carried the mosaic karyotype, 49,XX,+mar × 3[1]/47,XX,+mar[9]/46,XX[10], as described on the sSMC database (No.15-W-q14/7–1) [33]. In these two cases, the proportions of cells with 49 chromosomes were only 6% and 5%, respectively. However, this proportion was as high as 83% in the patient 1 of our study. A comparison of the phenotypes observed between our cases and the two previously reported individuals described above revealed that patient 1 in our study not only exhibited mental retardation but also cleft palate and hyperpigmentation. Thus, we speculated that the phenotypic severity is related to the level of mosaicism.

Idic(15) syndrome has a distinctive clinical phenotypes consisting of early central hypotonia, developmental delay, epilepsy, and autistic behavior (Table 2). Furthermore, partial idic(15) patients exhibit increased pigmentation, and the most affected individuals have subtle facial features, including a small button nose, down-slanting palpebral fissures, and low-set and/or posteriorly rotated ears [29]. Our patients possessed nearly all of the known phenotypes, except for epilepsy. The age of onset of epilepsy is usually between 6 months and 9 years old [31]. Therefore, these two patients could potentially develop epilepsy in the future.

**Table 2:** Overview of the clinical presentation of different numbers of idic(15) as reported in the literature

Clinical phenotype	Tetrasomy (*N* = 44) [7, 11–18]	Hexasomy (*N* = 8) [9, 20–23]	Octosomy (*N* = 3)^a^ [32, 33]
Mental retardation	33/44(75%)	6/8(75%)	3/3
Autism	10/44(22.7%)	2/8(25%)	_
Seizures	17/44(38.6%)	7/8(87.5%)	1/3
Aggressiveness	18/44(40.9%)	6/8(75%)	1/3
Sleep problems	4/44(9.1%)	_	1
Short stature	10/44(22.7%)	_	2/3
Language delay	17/44(38.6%)	6/8(75%)	1/3
Abnormal EEG	10/44(22.7%)	5/8(62.5%)	_
Abnormal MRI	6/44(13.6%)	2/8(25%)	normal
Dizziness	_	1/8(12.5%)	_
Mild facial anomalies	26/44(59.1%)	7/8(87.5%)	2/3
Strabismus	7/44(15.9%)	_	_
Nystagmus	1/44(2.3%)	_	1/3
Tympanitis/nervous deafness	16/44(36.4%)	2/8(25%)	normal
Cleft palate	_	1/8(12.5%)	1/3
Short neck	_	1/8(12.5%)	_
Low muscle tension	27/44(61.3%)	6/8(75%)	1/3
Hyperpigmentation	6/44(13.6%)	3/8(37.5%)	1/3
Bone disorders	4/44(9.1%)	1/8(12.5%)	_
Cryptorchidism	1/44(2.3%)	1/8(12.5%)	_
Joint abnormality	6/44(13.6%)	5/8(62.5%)	_
Exaggerated tendon reflex	1/44(2.3%)	1/8(12.5%)	Normal or _
Cannot walk	3/44(6.8%)	2/8(25%)	1/3

*N* the total number of patients, *_* not available or no phenotype, *EEG* electroencephalography, *MRI* magnetic resonance imaging

^a^the data were obtained from patients in our study, in the literature and in databases

A few of patients with Dup15q syndrome had presented hyperpigmentation [33]. In our study, both patients presented with skin hyperpigmentation, which was especially prominent in patient 1. The *P* gene, which is located in 15q(27,754,875–28,099,358), encodes a melanosomal transmembrane protein. Homozygous loss-of-function mutations in the *P* gene could cause hypopigmentation [OMIM#611409], while copy number increases in the *P* gene could cause hyperpigmentation [9, 12, 15, 16, 29]. Both patients were hyperpigmented, which is consistent with an increase in the *P* gene copy number. In patient 1, most of the body presented hyperpigmented, while patient 2 exhibited only partial hyperpigmentation, Additionally, the color in patient 1 was much deeper than that observed in patient 2, indicating that the phenotype associated with the *P* gene is dosage-sensitive. Furthermore, our patients displayed pigmentary mosaicism, which is consistent with a mosaic karyotype. Patient 2 presented with a hemangioma on her left leg while patient 1 had none. Nietzel et al. reported a girl with 48,XX,+mar × 2 who also presented with a hemangioma(5 × 5 cm) on her back [6]. However, we found no conclusive evidence indicating a correlation between idic(15) and hemangioma. The results of SNP analyses revealed that the duplication segment in patient 2 (9.7 Mb) was larger than in patient 1 (6.3 Mb). Therefore, we hypothesized that the reason for the hemangioma observed in patient 2 might be associated with the patient’s larger repeat segment.

Cook et al. firstly demonstrated that maternally inherited idic(15) is more likely than paternally inherited idic(15) to result in development impairments [10]. Idic(15) covers the PWACR area, which contains many imprinted genes. The genes that are maternally preferentially expressed in the PWACR area are specifically expressed in the brain, such as *UBE3A* and *ATP10A*. This area also contains bi-allelically expressed genes, such as GABA_A_ receptor subunit genes (*GABRB3, GABRA5,* and *GABRG3*), which may be overexpressed in patients with idic(15) in whom they can cause clinical effects [34, 35], including intellectual disabilities, autism and epilepsy [10, 13, 31, 35, 36]. In our study, we used DNA fingerprinting analysis to verify that the idic(15) of patient 1 was maternal. Thus, we think that the phenotype of patient 1 was associated with an increases in the maternal genes copy number.

A few of patients with Dup15q syndrome presented with cleft palate [9, 37]. In the present study, this symptom was also observed in patient 1. Erdogan et al. reported that candidate genes for cleft palate were located on chromosome 15 and included *GREM1* (32,717,974-32,745,107), *CX36* (34,751,032-34,754,965), and *MEIS2* (36,889,204-37,101,299) [38]. As these genes are outside of the range of the duplicated segment detected in patient 1, the cleft palate observed in patient 1 may be caused by another unknown gene.

## Conclusion

In summary, we report two cases with profound intellectual impairment, development delay and hyperpigmentation and identify a rare mosaic octosomy of 15q11-q13. We explored the relationship between phenotypes and karyotypes in these patients and verified that phenotypic severity was correlated with the level of mosaicism and the dosage effect of related genes in the proximal 15q region.

## Abbreviations

AS: Angelman syndrome
CNV: Copy number variation
FISH: Fluorescence in situ hybridization
PWACR: PWS/AS critical region
PWS: Prader-Willi syndrome (PWS)
SMC: Supernumerary marker chromosome
SNP: Single-nucleotide polymorphism
